# Intermingled cAMP, cGMP and calcium spatiotemporal dynamics in developing neuronal circuits

**DOI:** 10.3389/fncel.2014.00376

**Published:** 2014-11-13

**Authors:** Stefania Averaimo, Xavier Nicol

**Affiliations:** ^1^UMR_7210, Centre National de la Recherche ScientifiqueParis, France; ^2^UMR_S 968, Institut de la Vision, Sorbonne Universités, UPMC Univ Paris 06Paris, France; ^3^U968, Institut National de la Santé et de la Recherche MédicaleParis, France

**Keywords:** cAMP, cGMP, calcium, subcellular compartmentalization, kinetics, axon outgrowth, axon guidance, topographic maps

## Abstract

cAMP critically modulates the development of neuronal connectivity. It is involved in a wide range of cellular processes that require independent regulation. However, our understanding of how this single second messenger achieves specific modulation of the signaling pathways involved remains incomplete. The subcellular compartmentalization and temporal regulation of cAMP signals have recently been identified as important coding strategies leading to specificity. Dynamic interactions of this cyclic nucleotide with other second messenger including calcium and cGMP are critically involved in the regulation of spatiotemporal control of cAMP. Recent technical improvements of fluorescent sensors facilitate cAMP monitoring, whereas optogenetic tools permit spatial and temporal control of cAMP manipulations, all of which enabled the direct investigation of spatiotemporal characteristics of cAMP modulation in developing neurons. Focusing on neuronal polarization, neurotransmitter specification, axon guidance, and refinement of neuronal connectivity, we summarize herein the recent advances in understanding the features of cAMP signals and their dynamic interactions with calcium and cGMP involved in shaping the nervous system.

## Introduction

The development of nervous system connectivity is a multistage process that requires neuron specification and polarization, axon guidance and targeting, as well as refinement of synaptic connections. All stages require second messenger cascades involving cAMP. This cyclic nucleotide is required for the control of transcription (Pham et al., 2001; Root et al., 2008), involved in polarizing immature neurons (Shelly et al., 2010), promotes axon outgrowth (Roisen et al., 1972; Cai et al., 2001; Shewan et al., 2002; Corredor et al., 2012), and regulates the response of growing axons to guidance molecules (Song et al., 1997; Höpker et al., 1999; Nishiyama et al., 2003; Nicol et al., 2006b). Later during development, cAMP is crucial for the refinement of neuronal connectivity and the precise choice of synaptic partners (Welker et al., 1996; Ravary et al., 2003). It is also involved in a wide range of cellular processes that are not related to the development of neuronal connectivity including sugar and lipid metabolism. However, we still have a very partial understanding of how this second messenger can achieve specificity for each of its downstream pathways.

Although the influence of cAMP on the developing nervous system has been identified decades ago, the shape, duration or subcellular localization of its variations in living neurons is still elusive. The spatiotemporal attributes of cAMP signals might provide the bases of a code explaining the specificity of a given cAMP modulation for its proper downstream pathway. cAMP signaling is tightly related to two other second messengers, cGMP and calcium, and their intricate regulation contributes to enlarge the coding strategies leading to the activation of unique signaling pathways (Borodinsky and Spitzer, 2006; Zaccolo and Movsesian, 2007). A recent and expanding set of tools now enables a precise study of the features of cAMP signals. This includes a growing number of genetically-encoded FRET-based biosensors that have been improved over time (Zhang et al., 2001; Nikolaev et al., 2004; Ponsioen et al., 2004; Polito et al., 2013) and allow the monitoring of cAMP signals in living neurons. This toolbox is complemented with recently implemented optogenetic adenylyl cyclases (ACs—the cAMP-synthesizing enzyme), providing full spatio-temporal control of cAMP concentration in living neurons (Schröder-Lang et al., 2007; Ryu et al., 2010; Hong et al., 2011; Stierl et al., 2011). Said utilities have recently been applied to the study of nervous system connectivity, revealing some of the cAMP coding strategies in developing neurons.

## Mechanisms underlying the generation and control of spatiotemporal cAMP signals

**Figure 1:**
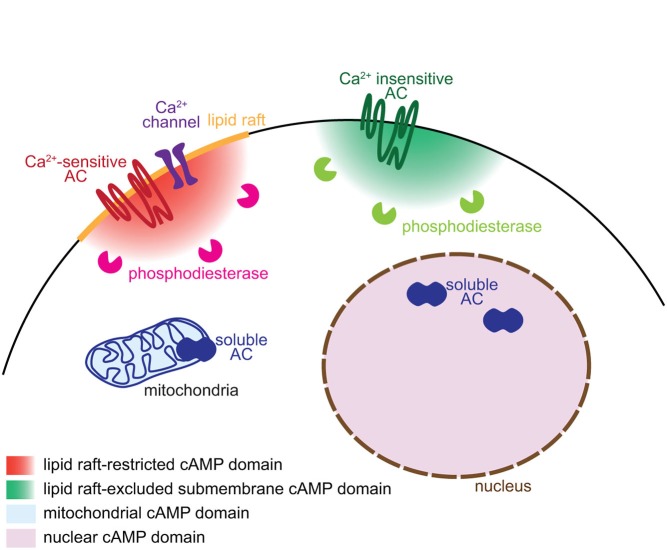
**Mechanisms underlying the generation of subcellular domains of cAMP**. Distinct subcellular localizations of adenylyl cyclases and phosphodiesterases enable the generation of subcellular domains of cAMP. Ca^2+^-sensitive transmembrane ACs and their regulating Ca^2+^ channels are restricted to lipid rafts whereas the Ca^2+^-independent ACs are excluded from this plasma membrane territory. Phosphodiesterases degrade cAMP close to its site of synthesis and limit the diffusion of the signal. The soluble AC is responsible of cAMP synthesis in discrete locations of the cell including the mitochondria and the nucleus.

The biological significance of cAMP signals during different steps of neuronal development relies on a tight control of both, their temporal and spatial features. Synthesis of cAMP is dependent on ACs, whereas the hydrolysis of cyclic nucleotides and extinction of the signal relies on phosphodiesterases (PDEs). The diversity of regulation and localization of ACs (10 isoforms) and PDEs (more than 40 isoforms) offers a wide range of combination to shape specific signals in response to distinct stimuli (Omori and Kotera, 2007; Willoughby and Cooper, 2007; Kleppisch, 2009). The cooperation between ACs and PDEs is crucial to control the time of onset of cAMP elevation and to limit the spatial and temporal expansion of the signal, forming the relevant coding strategies controlling specific activation of cAMP downstream effectors (Figure 1).

Different AC isoforms are targeted to distinct cellular microdomains, providing the basis of locally generated cAMP signals. sAC/AC10 is associated with intracellular compartments including mitochondria, centrosome and nucleus (Zippin et al., 2003; Valsecchi et al., 2014). The calcium-sensitive transmembrane ACs (AC1, 3, 5, 6 and 8) are tethered to lipid rafts, a cholesterol- and sphyngolipid-enriched compartment of the plasma membrane. In contrast, the calcium-independent ACs (AC2, 4, 7 and 9) are targeted to the plasma membrane but excluded from lipid rafts (Willoughby and Cooper, 2007; Figure 1). Targeting or exclusion of specific AC isoforms from lipid rafts might be the crucial molecular correlate of distinct cAMP concentrations and signaling in compartments of the plasma membrane (Depry et al., 2011). Lipid rafts compartmentalize cAMP signals induced by beta adrenergic receptors in cardiomyocytes (Agarwal et al., 2011). In platelets, compartmentalized cAMP signals are created by lipid rafts and the cytoskeleton (Raslan and Naseem, 2014). This plasma membrane compartment is of particular interest to understand the development of neuronal connectivity. Its integrity is crucial for BDNF-, netrin-1- and Sema3A-induced, but not glutamate-dependent turning of *X. laevis* axons (Guirland et al., 2004). Evaluating whether lipid raft-restricted cAMP signals are required for axon pathfinding presents a promising approach to deciphering the second-messenger codes involved in axon guidance. Precise localization of ACs is also crucial for the coordination between second messengers. Interaction between AC8 and the pore component of the store operated calcium channels ensures a dynamic and coordinated relation between cAMP and calcium (Willoughby et al., 2012).

The cAMP turnover, balanced by synthesis and degradation, requires a tight regulation. Like ACs, PDEs are crucial for the spatio-temporal control of cAMP signaling. Their distinct intracellular localization, kinetics and regulatory mechanisms enable to shape a wide range of signals that differ in their spatiotemporal features and upstream regulators. Compartmentalization of PDEs is responsible for simultaneously generating multiple and contiguous cAMP domains, even far from the site of synthesis (Terrin et al., 2006). According to this model, synthesized cAMP is free to diffuse. The specific activation of only a subset of its downstream pathways relies on the restricted subcellular localization of PDEs, acting locally as a cAMP sink to prevent the activation of downstream effectors (Figure 1). The cAMP-specific PDE4 family is crucial for this process in a wide number of cell types. In cardiomyocytes, the activation of PDE4D spatially limits the diffusion of cAMP, modulating cAMP-dependent protein kinase A (PKA) activation and the subsequent increase in calcium cycling required for contractile responses in the heart (Liu et al., 2012). In fibroblasts, PDE4B and PDE4D modulates cAMP concentration in discrete domains near the plasma membrane and are involved in distinct signaling pathways (Blackman et al., 2011). In neurons, the PDE4 family is involved in the functional compartmentation of cAMP, modulating the propagation of PKA activation from the plasma membrane to the nucleus (Castro et al., 2010; Vincent et al., 2012).

For ACs and PDEs to properly control the localization of cAMP signals, the targeting of these enzymes is tightly regulated. A-kinase anchoring proteins (AKAPs) are critical for this process. AKAP isoforms are targeted to distinct subcellular compartments and modulate the spatial spread of cAMP, binding at least some isoform of PDEs and ACs (Piggott et al., 2008; Willoughby et al., 2010; Delint-Ramirez et al., 2011; Terrin et al., 2012). In addition, AKAPs bind downstream effectors of cAMP including PKA and the cAMP-stimulated GDP exchange factors (Epacs), segregating distinct cAMP downstream pathways (Wong and Scott, 2004; McConnachie et al., 2006). Although to date there are only a few studies focusing on the spatial restriction of cAMP signals by AKAPs in developing neurons, these anchoring proteins have been extensively studied in other cell types. For instance, in airway smooth muscle cells, AKAPs modulates cAMP accumulation in response to β2-adrenergic agonists. PKA activation in turn phosphorylates PDE4, increasing its activity and reducing cAMP concentration in specific domains where AKAP proteins are localized (Horvat et al., 2012). In the nucleus, AKAPs have been proposed to control a PKA/PDE modulated cAMP signal different from that in the cytosol microdomain (Sample et al., 2012). Indeed, cAMP signaling at the plasma membrane followed by slow diffusion into the nucleus results into slow kinetics of PKA activity likely to be limited by the translocation of the catalytic domain of PKA from the cytosol to the nucleus. Indeed, PDEs keep the cAMP concentration in the nucleus too low to activate PKA. However, a direct activation of cAMP synthesis in the nucleus would result into fast kinetics of the nuclear PKA response. In this case, the spatio-temporal modulation of cAMP is responsible for a kinetically distinct activation of PKA, and the local negative regulator PDE4 contributes to establishing a local signaling threshold to convert spatial second messenger signals to temporal control of kinase activity. Finally, a dynamic and delicate control of cAMP signals has also been identified in the centrosome, a key subcellular structure for migration and cell cycle progression. In this subcellular domain, cAMP concentration is independent on cAMP levels in the cytosol and relies on PDE4D3 and PKA anchoring to AKAP 450 (Terrin et al., 2012).

## Early events: neuronal polarization and neurotransmitter specification

**Figure 2:**
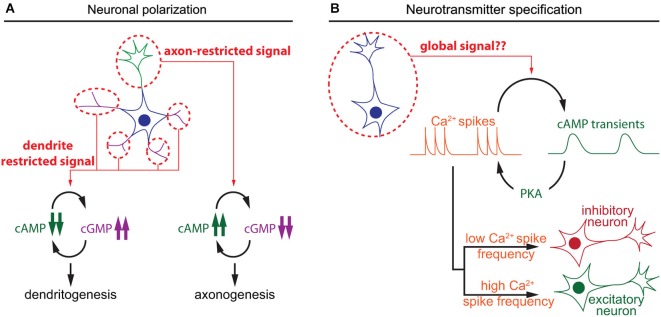
**Spatiotemporal dynamics of cAMP during neuronal polarization and neurotransmitter specification**. **(A)** Local interactions between cAMP and cGMP determine neurite maturation into axon or dendrite. High cAMP and low cGMP concentrations promote axonogenesis, whereas low cAMP and high cGMP lead to dentritogenesis. **(B)** In developing neurons, cAMP transients and calcium spikes are interdependent. The choice of expression of excitatory or an inhibitory neurotransmitter is modulated by the frequency of calcium spikes, which is regulated by the activity of a set of kinases including the cAMP effector PKA.

The polarization of postmitotic neurons leads to the segregation of two distinct subcellular compartments: a single axon and a somato-dendritic compartment including multiple dendrites (Arimura and Kaibuchi, 2007; Barnes et al., 2008). Axon/dendrite specification of undifferentiated neurites involves cyclic nucleotide signaling aiming at the development of a single axon in each neuron. The use of cAMP and cGMP reporters enabled the identification of local cyclic nucleotide signals regulating axon/dendrite specification. cAMP initiates a positive feedback involving phosphorylation of the serine/threonine kinase LKB1 (Liver Kinase B1) and its interactor STRAD (STE20-related adapter protein), leading to differentiation and stabilization of the axon (Shelly et al., 2007). In contrast, cGMP favors the differentiation of immature neurites into mature dentrites (Shelly et al., 2010). Local and distal interactions between these two second messengers create two subcellular compartments that define the polarity of the neuron. Local cAMP elevation at the tip of a neurite leads to a local reduction of cGMP concentration, and a distal cAMP suppression and cGMP elevation in the other neurites and vice versa (Shelly et al., 2010; Figure 2). This local modulation of cyclic nucleotide levels is observed in maturing neurons in response to extracellular cues that favor either axon or dendrite initiation like BDNF or Semaphorin3A respectively (Shelly et al., 2011), or by activation of GABA_B_ receptors (Bony et al., 2013). These observations emphasize the influence of local cyclic nucleotide signaling for axono- and dendritogenesis.

A second feature of neuronal differentiation is the specification of neurotransmitters. This is a crucial step in the development of the nervous system to ensure appropriate connectivity and to finely tune the balance between excitation and inhibition. In developing neurons, spontaneous calcium spikes play a critical role in the choice of neurotransmitter. The frequency of these calcium transients dictates the expression of an inhibitory or excitatory neurotransmitter. Expression of the hyperpolarizing potassium channel Kir2.1 suppresses calcium spikes in spinal *X. laevis* neurons and triggers the expression of the excitatory neurotransmitters machinery, including the glutamate vesicular transporter (VGluT) and choline acetyltransferase transporters (ChAT). In contrast, overexpression of the voltage gated sodium channel Na*_v_*1.2 increases calcium spike frequency and the number of the inhibitory GABAergic and glycinergic spinal neurons at the expense of VGluT and ChAT-expressing neurons, demonstrating that pattern calcium activity affects neuronal differentiation (Borodinsky et al., 2004). Metabotropic GABA_B_ receptors and group III metabotropic glutamate receptors regulate PKA activity, one of the main cAMP effectors, and modulate the generation of calcium spikes (Root et al., 2008). Interestingly, cAMP transients, rather than sustained elevation of this second messenger, are generated in *X. laevis* spinal neurons at the same developmental stage than calcium spikes that regulate neurotransmitter specification (Gorbunova and Spitzer, 2002). cAMP transients and calcium spikes are interdependent. Bursts of calcium spikes induce slow cAMP transients (3–7 min in young and older neurons respectively) and increasing cAMP concentration concomitantly increases the frequency of calcium spikes (Gorbunova and Spitzer, 2002; Figure 2). This reciprocal interaction between calcium and cAMP signal dynamics has been observed in a wide range of non-neuronal cell types (Landa et al., 2005; Borodinsky and Spitzer, 2006; Dyachok et al., 2006; Willoughby and Cooper, 2006). However, a possible direct relationship between cAMP transients and neurotransmitter specification has not been investigated. It would be of interest to evaluate if the frequency of cAMP transients influences neurotransmitter specification in developing neurons, like the frequency of calcium spikes.

## Axon outgrowth

**Figure 3:**
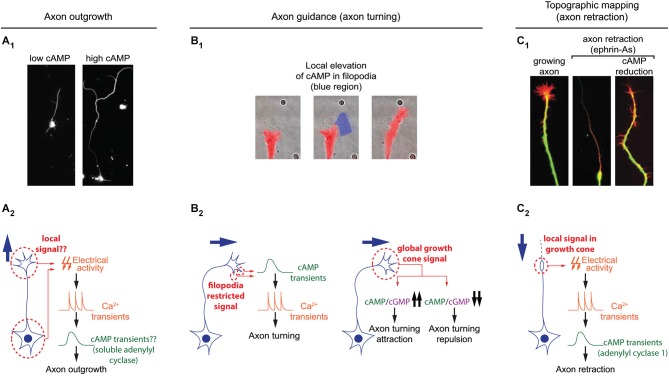
**Spatiotemporal dynamics of cAMP during axon outgrowth, axon guidance and topographic maps development. (A_1_)** Axon elongation is enhanced in cultured retinal ganglion cell with an increased cAMP concentration. **(A_2_)** Electrical activity and calcium transients modulates cAMP synthesis by the soluble adenylyl cyclase (sAC/AC10). Both signals cooperate to regulate axon outgrowth. It is still unclear whether or not this signal transduction pathway is spatiotemporally restricted. **(B_1_)** Transient and local cAMP synthesis by light-mediated activation of an optogenetic AC (within the blue region) is sufficient to induce axon turning. **(B_2_)** Brief cAMP signal in filopodia induces an increase in the frequency of filopodia-restricted Ca^2+^ transients and a change of direction in axon outgrowth. cAMP/cGMP ratio sets the polarity of netrin-1-induced axon guidance. A high cAMP/cGMP ratio leads to attraction whereas a low ratio converts attraction into repulsion. **(C_1_)** cAMP signaling perturbation (lack of AC1, pharmacological blockade of ACs, or PKA blockade) prevents the axon repellents ephrin-As to induce axon retraction. **(C_2_)** Transient electrical activity- and calcium-dependent modulation of cAMP is required in growth cones for the backward movement of axons when exposed to ephrin-As.

Once neurons are polarized, axons grow over long distances to reach their synaptic partners. Axon elongation is dependent on cAMP signaling and high concentration of this second messenger favors axon outgrowth (Roisen et al., 1972; Cai et al., 2001; Shewan et al., 2002; Corredor et al., 2012). In this process as well, cAMP signaling is tightly linked to calcium to regulate axon outgrowth. Transient calcium elevations, termed calcium waves, have been visualized in the growth cone of extending axons. Calcium waves cover the entire growth cone at once and their kinetics is relatively slow. The frequency is inversely proportional to the speed of axon outgrowth in a wide range of models: lower frequency of calcium transients correlates with a high rate of axonal growth, while a high frequency is observed in slow growing axons (Gomez et al., 1995; Gomez and Spitzer, 1999; Tang et al., 2003). For instance in the developing spinal cord of *X. laevis*, Rohon-Beard neurons, ventral motor neurons and ascending interneurons exhibit a low frequency of calcium transients and a rapid outgrowth. In contrast, the frequency of calcium transients is higher in the slow growing dorso-lateral ascending interneurons (Gomez and Spitzer, 1999). A direct link between cAMP signals modulating axon outgrowth and calcium waves in growth cones has not been investigated so far. However, cAMP transients have been detected in axonal growth cones exposed to netrin-1, a guidance molecule that modulates both, the direction and the speed of axon elongation. cAMP transients require extracellular calcium influx and coincide with an increased frequency of calcium waves, that are not dependent on transmembrane AC activity (Nicol et al., 2011; Figure 3). The transient change in cAMP suggests that a temporal control might contribute to the coding strategy of cAMP signals regulating axon outgrowth.

Only cAMP signals from a subset of ACs are able to modulate axon outgrowth. The synthesis of cAMP by soluble AC (sAC/AC10) but not transmembrane ACs (AC1 to 9) is required for the intrinsic ability of axons to grow (Corredor et al., 2012). sAC/AC10 is also critical for netrin-1-induced outgrowth (Wu et al., 2006), but does not contribute to the cAMP-dependent modulation of axon pathfinding by netrin-1 (Moore et al., 2008). This suggests that cAMP signals generated by different ACs are not identical, although the features allowing the distinction between diverse cAMP signals have not been identified. A likely hypothesis is that subcellular localization of signals would provide a code to link each cAMP signal to its appropriate downstream pathway. Indeed sAC/AC10 is restricted to intracellular compartments in non-neuronal cells (Zippin et al., 2003; Feng et al., 2006), whereas the other ACs are transmembrane proteins.

## Axon guidance

To reach their synaptic partners, axons follow stereotypic paths, guided by attractive and repulsive cues, which they encounter on the way to their final targets. cAMP signaling is a crucial modulator of axonal response to guidance molecules and enables the conversion of attractive cues into repulsive ones and vice versa (Ming et al., 1997; Song et al., 1997; Höpker et al., 1999; Murray et al., 2009). However, the spatio-temporal dynamics of cAMP signals regulating axon guidance have been investigated only recently. As during neurotransmitter specification and axon outgrowth, cAMP signaling involved in axon pathfinding is linked to calcium. Calcium signaling is critical for axonal response to guidance molecules (Hong et al., 2000; Nishiyama et al., 2003; Li et al., 2009). In addition to slow calcium waves regulating axon outgrowth, fast calcium transients restricted to filopodia have been visualized in developing axons (Gomez et al., 2001). An asymmetric frequency of calcium transients in filopodia from both sides of the growth cone is able to orient axon outgrowth (Gomez et al., 2001; Robles et al., 2003). Guidance molecules such as netrin-1 modulate the frequency of filopodial calcium transients. Transient elevations of cAMP precede and are required for the increase in frequency of local filopodial calcium transients (Nicol et al., 2011), making cAMP crucial to orient netrin-1-induced axon outgrowth. Although netrin-1 exposure also drives an increase in cAMP concentration in the central domain of the growth cone, this signal is not involved in the regulation of axon growth direction (Nicol et al., 2011). This suggests that axon guidance molecules can induce cAMP synthesis in a restricted area of the growth cone for a limited duration. This idea is supported by observations of axon turning generated by exogenous spatially- and temporally-restricted cAMP signals *in vitro* (Munck et al., 2004; Nicol et al., 2011). *In vivo*, transient cAMP elevations are sufficient to restore outgrowth of commissural axons towards the ventral midline of the spinal cord when DCC-dependent netrin-1 signaling is blocked. In contrast, sustained increase or decrease of cAMP concentration leads to abnormal trajectories of spinal commissural axons in *X. laevis* (Nicol et al., 2011), suggesting that spatio-temporal features of cAMP signals are crucial for the modulation of axonal pathfinding (Figure 3).

The regulation of axon pathfinding by cAMP signaling tightly controlled in space and time is most likely not the only cAMP-dependent process regulating axon guidance. Axon turning can be modulated by sustained and global manipulations of PKA activity that are sufficient to modify the amplitude of imposed fast and local calcium transients dependent on ryanodine receptors (Ooashi et al., 2005). The resting cAMP concentration is tuned by adhesion molecules such as laminin that also regulate axon guidance (Höpker et al., 1999; Ooashi et al., 2005). In addition, cAMP interacts with cGMP signaling, often counteracting its effects. Whereas high cAMP concentration favors attraction, cGMP is associated with axonal repulsion. A high cAMP/cGMP ratio in spinal axons leads to attraction by netrin-1, whereas a low ratio converts this attraction into repulsion. This highlights the relevance of relative cAMP levels compared to cGMP rather than an absolute concentration of one or the other cyclic nucleotide (Nishiyama et al., 2003; Figure 3). The antagonizing effect of cAMP and cGMP might be due to their opposite regulation of common downstream effectors. Both second messengers modulate the amplitude of the ryanodine receptor-dependent local calcium transient. The cAMP/PKA pathway promotes ryanodine receptor-mediated calcium induced calcium release, whereas cGMP and its downstream effector protein kinase G (PKG) reduces the ryanodine receptor-dependent mobilization of internal calcium stores (Ooashi et al., 2005; Tojima et al., 2009). Simultaneous imaging of cAMP and cGMP in growing axons emphasized a temporally correlated regulation of cAMP and cGMP in growth cones. As during axonogenesis, an increase of cAMP correlates with a reduction in cGMP concentration (Kobayashi et al., 2013). The mechanisms underlying these opposite modulations are unclear. In other systems, inverse regulation of both cyclic nucleotides are related to PDE activity (Shelly et al., 2010; Polito et al., 2013). So far, not much attention has been attributed to evaluating the impact of these cAMP/cGMP degrading enzymes, which might be crucial players in cyclic nucleotide oscillations in axon guidance.

## Axon branching and targeting (topographic maps)

Once axons have reached their targets, dense terminal axonal arbors develop. The precise positioning of this termination zone is tightly controlled and crucial for appropriate wiring of the nervous system. This process has been extensively studied in sensory systems, in which projections are topographically organized. Axonal arbors are organized in their target regions in correlation with the information they carry. For instance, axons originating from neighboring retinal ganglion cells arborize in neighboring areas in their targets. Similarly, olfactory neurons expressing the same olfactory receptor target the same region of the olfactory bulb. cAMP signaling is crucial for the development of topographic maps. Among the ACs tested, only two are required for topographic map formation: AC1 in the visual and somatosensory systems (Welker et al., 1996; Ravary et al., 2003; Nicol et al., 2006a), and AC3 in the olfactory projections (Wong et al., 2000; Col et al., 2007; Zou et al., 2007). In AC1 and AC3 knock-out mice, ectopic axonal branches fail to be pruned during development resulting in larger termination zones. In contrast, AC1 and AC3 are not involved in axon outgrowth, a process requiring sAC/AC10 (Wu et al., 2006; Corredor et al., 2012). The distinct roles of each AC suggest that they generate cAMP signals with unique features.

AC1-dependent cAMP signaling involved in the development of topographic maps requires calcium. Retinal axons lacking this calcium-activated AC (Fagan et al., 2000) or exposed to calcium-free extracellular medium show the same phenotype when exposed to the axon repellent ephrin-As: in both conditions axons fail to retract (Nicol et al., 2006b, 2007). This reduced retraction is likely to contribute to the impaired axonal pruning seen in AC1^−/−^ mice since ephrin-As are crucial for the development of retinal topography (Frisén et al., 1998; Feldheim et al., 2000). Ephrin-A-induced retraction also requires electrical activity. In absence of electrical activity, short and repeated cAMP pulses in growth cones are sufficient to rescue axonal retraction. In contrast, global and sustained elevation of cAMP does not rescue axon retraction. Sustained high or low cAMP concentration is sufficient to prevent ephrin-A-induced repulsion, suggesting that the temporal features of cAMP signaling is crucial for axonal retraction and the development of topographic maps (Nicol et al., 2007; Figure 3).

AC3 is required for the correct antero-posterior position in terminal zones of olfactory projections and for the convergence of olfactory axons expressing the same olfactory receptor into the same glomerulus (Wong et al., 2000; Col et al., 2007; Zou et al., 2007). The cAMP-dependent signaling pathways involved differ in these two developmental processes, only one of them interacting with calcium signaling. AC3-dependent cAMP signaling regulates the expression of the axon guidance molecule semaphorin-3A and its receptor neuropilin-1 to modulate the position of axonal arbors along the antero-posterior axis in a CREB-dependent mechanism (Imai et al., 2006). In contrast, AC3-dependent activation of the cyclic nucleotide gated calcium channel α2 is required for the development of glomeruli receiving axons expressing the same olfactory receptor (Serizawa et al., 2006). The spatiotemporal analysis of cAMP modulation in developing olfactory neurons revealed that activation of olfactory receptors at axonal growth cones induces a local increase of cAMP and calcium, followed by the nuclear translocation of the catalytic subunit of PKA (Maritan et al., 2009). Exposure of olfactory axon terminals to odorants also leads to a local cAMP- and calcium-dependent increase of cGMP (Pietrobon et al., 2011). Activation of downstream cGMP signaling is sufficient but not necessary to induce CREB phosphorylation in the nucleus of olfactory neurons (Pietrobon et al., 2011), suggesting that cGMP might be involved in the modulation of gene expression during olfactory map development.

## Activity-dependent axonal and synaptic competition

**Figure 4:**
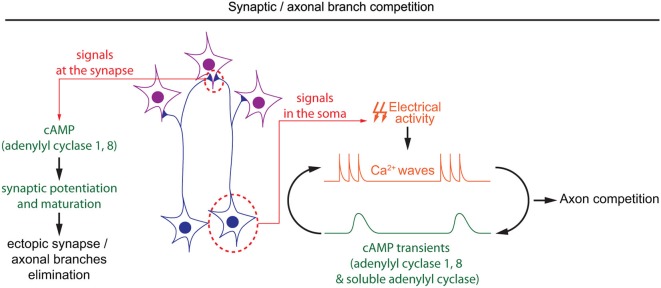
**Spatiotemporal dynamics of cAMP during developmental pruning of ectopic axonal branches, and synaptic competition**. cAMP is required in two subcellular compartments during the synaptic refinement of neuronal projections. In the soma of retinal ganglion cells, cAMP oscillations and spontaneous calcium waves are interdependent. Spontaneous calcium waves are required for competition between axons in their targets. In the synapses, cAMP signaling mediated by adenylyl cyclases 1 and 8 is involved in synaptic potentiation and hebbian mechanisms, which are crucial to eliminate misplaced synapses.

Once axonal branches are positioned in their targets, they carefully choose their synaptic partners, often through an axonal or synaptic competition process. cAMP has a crucial role in modulating this competition that represents a critical process for the development of a properly organized neuronal connectivity (Luo and O’Leary, 2005; Luo and Flanagan, 2007). Competition is dependent on electrical activity, and its regulation by cAMP often relates to spontaneous activity generated during development (Penn et al., 1998; Stellwagen and Shatz, 2002; Zhang et al., 2012; Furman et al., 2013). In the developing retina, spontaneous calcium waves appear prior to vision. The frequency and propagation of these calcium waves are modulated by cAMP, with a higher frequency and a propagation over longer distances when cAMP is increased (Stellwagen et al., 1999; Stellwagen and Shatz, 2002). Interactions between cAMP and calcium waves in the developing retina are bidirectional: depolarization-induced calcium influx is sufficient to produce brief cAMP elevation, and cAMP transients are spontaneously generated in post-natal retinal ganglion cells (Dunn et al., 2006; Figure 4). Like the cAMP elevations observed in *X. laevis* spinal neurons, spontaneous cAMP / PKA activity transients in the developing retina are observed upon long but not short bursts of electrical activity (Gorbunova and Spitzer, 2002; Dunn et al., 2006). Surprisingly, the genetic removal of AC1, the main calcium-stimulated AC expressed in retinal ganglion cells, is not sufficient to perturb depolarization-induced cAMP elevations. In AC1/AC8 double knock-out mice, cAMP transients only exhibit a reduced amplitude and are abolished only with pharmacological blockade of both, transmembrane (AC1 to 9) and soluble (sAC/AC10) ACs, suggesting that several ACs cooperate to generate brief cAMP / PKA activity transients in the retina (Dunn et al., 2009).

During development, cAMP-dependent spontaneous electrical activity in the retina is crucial for the precise organization of retinal axons in their targets. Retinal axons from each eye arborize in non-overlapping regions of their targets in a competition-dependent mechanism. When retinal calcium waves are perturbed, the normally well segregated territories occupied by axons from each eye overlap (Rossi et al., 2001; Muir-Robinson et al., 2002). Although AC1 is not required for the generation of cAMP transients or calcium waves in the developing retina (Dunn et al., 2009; Dhande et al., 2012), its genetic removal prevents the segregation of retinal projections in eye-specific territories in the dorso-lateral geniculate nucleus and the superior colliculus (Ravary et al., 2003; Nicol et al., 2006a), revealing that other cAMP signals are dependent on this particular AC. The stimulation of AC1 by calcium influx (Fagan et al., 2000), and the phenotypic similarity between AC1^−/−^ mice and models displaying altered spontaneous retinal waves or impaired electrical activity suggests that AC1 and calcium waves are part of the same signaling pathway regulating the development of eye-specific territories (Rossi et al., 2001; Muir-Robinson et al., 2002; Dhande et al., 2011). Converging evidence indicates that the deficit in the segregation of eye-specific territories in AC1^−/−^ mice is dependent on AC activity in retinal ganglion cells but not their targets (Nicol et al., 2006b; Dhande et al., 2012). Since cAMP signaling is not affected in the soma of retinal ganglion cells in AC1-deficient mice, it is probable that cAMP signaling restricted to the axonal/synaptic compartment plays a role in the competition leading to segregation of eye-specific territories. AC1 is required for synaptic maturation and potentiation, a process required for the refinement of retinal connections (Lu et al., 2006; Iwasato et al., 2008; Figure 4). However, the features of cAMP signaling required for synaptic competition need to be characterized in more detail to improve our understanding of the signaling code involved in synaptic competition.

## Conclusion

cAMP is involved in a wide range of processes influencing the development of nervous system connectivity. We summarize the recent progress in investigating the specific modulation of these multiple processes by spatio-temporally distinct cAMP signals. Temporal control of cAMP signals in subcellular compartments is a potential coding strategy to translate distinct cAMP signals into activation of specific downstream pathways in developing neurons. Crosstalk with other second messenger systems (cGMP and calcium) are of special importance to gain new insight into the spatiotemporal control of cAMP signals. Pursuing the analysis of these coding strategies will provide a better understanding of the regulatory second messenger networks shaping neuronal connectivity.

## Conflict of interest statement

The authors declare that the research was conducted in the absence of any commercial or financial relationships that could be construed as a potential conflict of interest.

## References

[B1] Agarwal S. R. MacDougall D. A. Tyser R. Pugh S. D. Calaghan S. C. Harvey R. D. (2011). Effects of cholesterol depletion on compartmentalized cAMP responses in adult cardiac myocytes. J. Mol. Cell. Cardiol. 50, 500–509. 10.1016/j.yjmcc.2010.11.015 21115018 PMC3049871

[B2] Arimura N. Kaibuchi K. (2007). Neuronal polarity: from extracellular signals to intracellular mechanisms. Nat. Rev. Neurosci. 8, 194–205. 10.1038/nrn2056 17311006

[B3] Barnes A. P. Solecki D. Polleux F. (2008). New insights into the molecular mechanisms specifying neuronal polarity in vivo. Curr. Opin. Neurobiol. 18, 44–52. 10.1016/j.conb.2008.05.003 18514505 PMC2488405

[B4] Blackman B. E. Horner K. Heidmann J. Wang D. Richter W. Rich T. C.. (2011). PDE4D and PDE4B function in distinct subcellular compartments in mouse embryonic fibroblasts. J. Biol. Chem. 286, 12590–12601. 10.1074/jbc.m110.203604 21288894 PMC3069460

[B5] Bony G. Szczurkowska J. Tamagno I. Shelly M. Contestabile A. Cancedda L. (2013). Non-hyperpolarizing GABAB receptor activation regulates neuronal migration and neurite growth and specification by cAMP/LKB1. Nat. Commun. 4:1800. 10.1038/ncomms2820 23653212

[B7] Borodinsky L. N. Root C. M. Cronin J. A. Sann S. B. Gu X. Spitzer N. C. (2004). Activity-dependent homeostatic specification of transmitter expression in embryonic neurons. Nature 429, 523–530. 10.1038/nature02518 15175743

[B6] Borodinsky L. N. Spitzer N. C. (2006). Second messenger pas de deux: the coordinated dance between calcium and cAMP. Sci. STKE 2006:pe22. 10.1126/stke.3362006pe22 16720840

[B8] Cai D. Qiu J. Cao Z. McAtee M. Bregman B. S. Filbin M. T. (2001). Neuronal cyclic AMP controls the developmental loss in ability of axons to regenerate. J. Neurosci. 21, 4731–4739. 11425900 10.1523/JNEUROSCI.21-13-04731.2001 PMC6762375

[B9] Castro L. R. V. Gervasi N. Guiot E. Cavellini L. Nikolaev V. O. Paupardin-Tritsch D.. (2010). Type 4 phosphodiesterase plays different integrating roles in different cellular domains in pyramidal cortical neurons. J. Neurosci. 30, 6143–6151. 10.1523/jneurosci.5851-09.2010 20427672 PMC6632585

[B10] Col J. A. D. Matsuo T. Storm D. R. Rodriguez I. (2007). Adenylyl cyclase-dependent axonal targeting in the olfactory system. Development 134, 2481–2489. 10.1242/dev.006346 17537788

[B11] Corredor R. G. Trakhtenberg E. F. Pita-Thomas W. Jin X. Hu Y. Goldberg J. L. (2012). Soluble adenylyl cyclase activity is necessary for retinal ganglion cell survival and axon growth. J. Neurosci. 32, 7734–7744. 10.1523/jneurosci.5288-11.2012 22649251 PMC3372574

[B12] Delint-Ramirez I. Willoughby D. Hammond G. V. R. Ayling L. J. Cooper D. M. F. (2011). Palmitoylation targets AKAP79 protein to lipid rafts and promotes its regulation of calcium-sensitive adenylyl cyclase type 8. J. Biol. Chem. 286, 32962–32975. 10.1074/jbc.m111.243899 21771783 PMC3190942

[B13] Depry C. Allen M. D. Zhang J. (2011). Visualization of PKA activity in plasma membrane microdomains. Mol. Biosyst. 7, 52–58. 10.1039/c0mb00079e 20838685

[B14] Dhande O. S. Bhatt S. Anishchenko A. Elstrott J. Iwasato T. Swindell E. C.. (2012). Role of adenylate cyclase 1 in retinofugal map development. J. Comp. Neurol. 520, 1562–1583. 10.1002/cne.23000 22102330 PMC3563095

[B15] Dhande O. S. Hua E. W. Guh E. Yeh J. Bhatt S. Zhang Y.. (2011). Development of single retinofugal axon arbors in normal and β2 knock-out mice. J. Neurosci. 31, 3384–3399. 10.1523/JNEUROSCI.4899-10.2011 21368050 PMC3060716

[B16] Dunn T. A. Storm D. R. Feller M. B. (2009). Calcium-dependent increases in protein kinase-A activity in mouse retinal ganglion cells are mediated by multiple adenylate cyclases. PLoS One 4:e7877. 10.1371/journal.pone.0007877 19924297 PMC2774513

[B17] Dunn T. A. Wang C.-T. Colicos M. A. Zaccolo M. DiPilato L. M. Zhang J.. (2006). Imaging of cAMP levels and protein kinase A activity reveals that retinal waves drive oscillations in second-messenger cascades. J. Neurosci. 26, 12807–12815. 10.1523/jneurosci.3238-06.2006 17151284 PMC2931275

[B18] Dyachok O. Isakov Y. Sågetorp J. Tengholm A. (2006). Oscillations of cyclic AMP in hormone-stimulated insulin-secreting beta-cells. Nature 439, 349–352. 10.1038/nature04410 16421574

[B19] Fagan K. A. Graf R. A. Tolman S. Schaack J. Cooper D. M. (2000). Regulation of a Ca2+-sensitive adenylyl cyclase in an excitable cell. Role of voltage-gated versus capacitative Ca2+ entry. J. Biol. Chem. 275, 40187–40194. 10.1074/jbc.m006606200 11010970

[B20] Feldheim D. A. Kim Y. I. Bergemann A. D. Frisén J. Barbacid M. Flanagan J. G. (2000). Genetic analysis of ephrin-A2 and ephrin-A5 shows their requirement in multiple aspects of retinocollicular mapping. Neuron 25, 563–574. 10.1016/s0896-6273(00)81060-0 10774725

[B21] Feng Q. Zhang Y. Li Y. Liu Z. Zuo J. Fang F. (2006). Two domains are critical for the nuclear localization of soluble adenylyl cyclase. Biochimie 88, 319–328. 10.1016/j.biochi.2005.09.003 16309816

[B22] Frisén J. Yates P. A. McLaughlin T. Friedman G. C. O’Leary D. D. Barbacid M. (1998). Ephrin-A5 (AL-1/RAGS) is essential for proper retinal axon guidance and topographic mapping in the mammalian visual system. Neuron 20, 235–243. 10.1016/s0896-6273(00)80452-3 9491985

[B23] Furman M. Xu H.-P. Crair M. C. (2013). Competition driven by retinal waves promotes morphological and functional synaptic development of neurons in the superior colliculus. J. Neurophysiol. 110, 1441–1454. 10.1152/jn.01066.2012 23741047 PMC3763158

[B25] Gomez T. M. Robles E. Poo M. Spitzer N. C. (2001). Filopodial calcium transients promote substrate-dependent growth cone turning. Science 291, 1983–1987. 10.1126/science.1056490 11239161

[B26] Gomez T. M. Snow D. M. Letourneau P. C. (1995). Characterization of spontaneous calcium transients in nerve growth cones and their effect on growth cone migration. Neuron 14, 1233–1246. 10.1016/0896-6273(95)90270-8 7605634

[B24] Gomez T. M. Spitzer N. C. (1999). In vivo regulation of axon extension and pathfinding by growth-cone calcium transients. Nature 397, 350–355. 10.1038/16927 9950427

[B27] Gorbunova Y. V. Spitzer N. C. (2002). Dynamic interactions of cyclic AMP transients and spontaneous Ca2+ spikes. Nature 418, 93–96. 10.1038/nature00835 12097913

[B28] Guirland C. Suzuki S. Kojima M. Lu B. Zheng J. Q. (2004). Lipid rafts mediate chemotropic guidance of nerve growth cones. Neuron 42, 51–62. 10.1016/s0896-6273(04)00157-6 15066264

[B30] Hong K. Nishiyama M. Henley J. Tessier-Lavigne M. Poo M. (2000). Calcium signalling in the guidance of nerve growth by netrin-1. Nature 403, 93–98. 10.1038/47507 10638760

[B29] Hong K. P. Spitzer N. C. Nicol X. (2011). Improved molecular toolkit for cAMP studies in live cells. BMC Res. Notes 4:241. 10.1186/1756-0500-4-241 21774821 PMC3152522

[B31] Höpker V. H. Shewan D. Tessier-Lavigne M. Poo M. Holt C. (1999). Growth-cone attraction to netrin-1 is converted to repulsion by laminin-1. Nature 401, 69–73. 10.1038/43441 10485706

[B32] Horvat S. J. Deshpande D. A. Yan H. Panettieri R. A. Codina J. DuBose T. D. Jr.. (2012). A-kinase anchoring proteins regulate compartmentalized cAMP signaling in airway smooth muscle. FASEB J. 26, 3670–3679. 10.1096/fj.11-201020 22649031 PMC3425821

[B33] Imai T. Suzuki M. Sakano H. (2006). Odorant receptor-derived cAMP signals direct axonal targeting. Science 314, 657–661. 10.1126/science.1131794 16990513

[B34] Iwasato T. Inan M. Kanki H. Erzurumlu R. S. Itohara S. Crair M. C. (2008). Cortical adenylyl cyclase 1 is required for thalamocortical synapse maturation and aspects of layer IV barrel development. J. Neurosci. 28, 5931–5943. 10.1523/JNEUROSCI.0815-08.2008 18524897 PMC2733830

[B35] Kleppisch T. (2009). Phosphodiesterases in the central nervous system. Handb. Exp. Pharmacol. 191, 71–92. 10.1007/978-3-540-68964-5_5 19089326

[B36] Kobayashi T. Nagase F. Hotta K. Oka K. (2013). Crosstalk between second messengers predicts the motility of the growth cone. Sci. Rep. 3:3118. 10.1038/srep03118 24176909 PMC3814829

[B37] Landa L. R. Jr. Harbeck M. Kaihara K. Chepurny O. Kitiphongspattana K. Graf O.. (2005). Interplay of Ca2+ and cAMP signaling in the insulin-secreting MIN6 beta-cell line. J. Biol. Chem. 280, 31294–31302. 10.1074/jbc.m505657200 15987680 PMC3508785

[B38] Li L. Hutchins B. I. Kalil K. (2009). Wnt5a induces simultaneous cortical axon outgrowth and repulsive axon guidance through distinct signaling mechanisms. J. Neurosci. 29, 5873–5883. 10.1523/jneurosci.0183-09.2009 19420254 PMC2697037

[B39] Liu S. Li Y. Kim S. Fu Q. Parikh D. Sridhar B.. (2012). Phosphodiesterases coordinate cAMP propagation induced by two stimulatory G protein-coupled receptors in hearts. Proc. Natl. Acad. Sci. U S A 109, 6578–6583. 10.1073/pnas.1117862109 22493261 PMC3340097

[B40] Lu H.-C. Butts D. A. Kaeser P. S. She W.-C. Janz R. Crair M. C. (2006). Role of efficient neurotransmitter release in barrel map development. J. Neurosci. 26, 2692–2703. 10.1523/jneurosci.3956-05.2006 16525048 PMC6675166

[B41] Luo L. Flanagan J. G. (2007). Development of continuous and discrete neural maps. Neuron 56, 284–300. 10.1016/j.neuron.2007.10.014 17964246

[B42] Luo L. O’Leary D. D. M. (2005). Axon retraction and degeneration in development and disease. Annu. Rev. Neurosci. 28, 127–156. 10.1146/annurev.neuro.28.061604.135632 16022592

[B43] Maritan M. Monaco G. Zamparo I. Zaccolo M. Pozzan T. Lodovichi C. (2009). Odorant receptors at the growth cone are coupled to localized cAMP and Ca2+ increases. Proc. Natl. Acad. Sci. U S A 106, 3537–3542. 10.1073/pnas.0813224106 19218439 PMC2642662

[B44] McConnachie G. Langeberg L. K. Scott J. D. (2006). AKAP signaling complexes: getting to the heart of the matter. Trends Mol. Med. 12, 317–323. 10.1016/j.molmed.2006.05.008 16809066

[B45] Ming G. L. Song H. J. Berninger B. Holt C. E. Tessier-Lavigne M. Poo M. M. (1997). cAMP-dependent growth cone guidance by netrin-1. Neuron 19, 1225–1235. 10.1016/s0896-6273(00)80414-6 9427246

[B46] Moore S. W. Lai Wing Sun K. Xie F. Barker P. A. Conti M. Kennedy T. E. (2008). Soluble adenylyl cyclase is not required for axon guidance to netrin-1. J. Neurosci. 28, 3920–3924. 10.1523/jneurosci.0547-08.2008 18400890 PMC6670467

[B47] Muir-Robinson G. Hwang B. J. Feller M. B. (2002). Retinogeniculate axons undergo eye-specific segregation in the absence of eye-specific layers. J. Neurosci. 22, 5259–5264. 12097474 10.1523/JNEUROSCI.22-13-05259.2002 PMC6758243

[B48] Munck S. Bedner P. Bottaro T. Harz H. (2004). Spatiotemporal properties of cytoplasmic cyclic AMP gradients can alter the turning behaviour of neuronal growth cones. Eur. J. Neurosci. 19, 791–797. 10.1111/j.0953-816x.2004.03118.x 15009126

[B49] Murray A. J. Tucker S. J. Shewan D. A. (2009). cAMP-dependent axon guidance is distinctly regulated by Epac and protein kinase A. J. Neurosci. 29, 15434–15444. 10.1523/jneurosci.3071-09.2009 20007468 PMC6666109

[B50] Nicol X. Bennis M. Ishikawa Y. Chan G. C.-K. Repérant J. Storm D. R.. (2006a). Role of the calcium modulated cyclases in the development of the retinal projections. Eur. J. Neurosci. 24, 3401–3414. 10.1111/j.1460-9568.2006.05227.x 17229090

[B51] Nicol X. Hong K. P. Spitzer N. C. (2011). Spatial and temporal second messenger codes for growth cone turning. Proc. Natl. Acad. Sci. U S A 108, 13776–13781. 10.1073/pnas.1100247108 21795610 PMC3158175

[B52] Nicol X. Muzerelle A. Rio J. P. Métin C. Gaspar P. (2006b). Requirement of adenylate cyclase 1 for the ephrin-A5-dependent retraction of exuberant retinal axons. J. Neurosci. 26, 862–872. 10.1523/jneurosci.3385-05.2006 16421306 PMC6675379

[B53] Nicol X. Voyatzis S. Muzerelle A. Narboux-Nême N. Südhof T. C. Miles R.. (2007). cAMP oscillations and retinal activity are permissive for ephrin signaling during the establishment of the retinotopic map. Nat. Neurosci. 10, 340–347. 10.1038/nn1842 17259982

[B54] Nikolaev V. O. Bünemann M. Hein L. Hannawacker A. Lohse M. J. (2004). Novel single chain cAMP sensors for receptor-induced signal propagation. J. Biol. Chem. 279, 37215–37218. 10.1074/jbc.C400302200 15231839

[B55] Nishiyama M. Hoshino A. Tsai L. Henley J. R. Goshima Y. Tessier-Lavigne M.. (2003). Cyclic AMP/GMP-dependent modulation of Ca2+ channels sets the polarity of nerve growth-cone turning. Nature 423, 990–995. 10.1038/nature01751 12827203

[B56] Omori K. Kotera J. (2007). Overview of PDEs and their regulation. Circ. Res. 100, 309–327. 10.1161/01.res.0000256354.95791.f1 17307970

[B57] Ooashi N. Futatsugi A. Yoshihara F. Mikoshiba K. Kamiguchi H. (2005). Cell adhesion molecules regulate Ca2+-mediated steering of growth cones via cyclic AMP and ryanodine receptor type 3. J. Cell Biol. 170, 1159–1167. 10.1083/jcb.200503157 16172206 PMC2171540

[B58] Penn A. A. Riquelme P. A. Feller M. B. Shatz C. J. (1998). Competition in retinogeniculate patterning driven by spontaneous activity. Science 279, 2108–2112. 10.1126/science.279.5359.2108 9516112

[B59] Pham T. A. Rubenstein J. L. Silva A. J. Storm D. R. Stryker M. P. (2001). The CRE/CREB pathway is transiently expressed in thalamic circuit development and contributes to refinement of retinogeniculate axons. Neuron 31, 409–420. 10.1016/s0896-6273(01)00381-6 11516398

[B60] Pietrobon M. Zamparo I. Maritan M. Franchi S. A. Pozzan T. Lodovichi C. (2011). Interplay among cGMP, cAMP and Ca2+ in living olfactory sensory neurons in vitro and in vivo. J. Neurosci. 31, 8395–8405. 10.1523/jneurosci.6722-10.2011 21653844 PMC6623327

[B61] Piggott L. A. Bauman A. L. Scott J. D. Dessauer C. W. (2008). The A-kinase anchoring protein Yotiao binds and regulates adenylyl cyclase in brain. Proc. Natl. Acad. Sci. U S A 105, 13835–13840. 10.1073/pnas.0712100105 18772391 PMC2544540

[B62] Polito M. Klarenbeek J. Jalink K. Paupardin-Tritsch D. Vincent P. Castro L. R. V. (2013). The NO/cGMP pathway inhibits transient cAMP signals through the activation of PDE2 in striatal neurons. Front. Cell. Neurosci. 7:211. 10.3389/fncel.2013.00211 24302895 PMC3831346

[B63] Ponsioen B. Zhao J. Riedl J. Zwartkruis F. van der Krogt G. Zaccolo M.. (2004). Detecting cAMP-induced Epac activation by fluorescence resonance energy transfer: Epac as a novel cAMP indicator. EMBO Rep. 5, 1176–1180. 10.1038/sj.embor.7400290 15550931 PMC1299185

[B64] Raslan Z. Naseem K. M. (2014). Compartmentalisation of cAMP-dependent signalling in blood platelets: the role of lipid rafts and actin polymerisation. Platelets, [Epub ahead of print]. 1–9. 10.3109/09537104.2014.916792 24832788

[B65] Ravary A. Muzerelle A. Hervé D. Pascoli V. Ba-Charvet K. N. Girault J.-A.. (2003). Adenylate cyclase 1 as a key actor in the refinement of retinal projection maps. J. Neurosci. 23, 2228–2238. 12657682 10.1523/JNEUROSCI.23-06-02228.2003 PMC6742000

[B66] Robles E. Huttenlocher A. Gomez T. M. (2003). Filopodial calcium transients regulate growth cone motility and guidance through local activation of calpain. Neuron 38, 597–609. 10.1016/s0896-6273(03)00260-5 12765611

[B67] Roisen F. J. Murphy R. A. Pichichero M. E. Braden W. G. (1972). Cyclic adenosine monophosphate stimulation of axonal elongation. Science 175, 73–74. 10.1126/science.175.4017.73 4332820

[B68] Root C. M. Velázquez-Ulloa N. A. Monsalve G. C. Minakova E. Spitzer N. C. (2008). Embryonically expressed GABA and glutamate drive electrical activity regulating neurotransmitter specification. J. Neurosci. 28, 4777–4784. 10.1523/jneurosci.4873-07.2008 18448654 PMC3318922

[B69] Rossi F. M. Pizzorusso T. Porciatti V. Marubio L. M. Maffei L. Changeux J.-P. (2001). Requirement of the nicotinic acetylcholine receptor β2 subunit for the anatomical and functional development of the visual system. Proc. Natl. Acad. Sci. U S A 98, 6453–6458. 10.1073/pnas.101120998 11344259 PMC33489

[B70] Ryu M.-H. Moskvin O. V. Siltberg-Liberles J. Gomelsky M. (2010). Natural and engineered photoactivated nucleotidyl cyclases for optogenetic applications. J. Biol. Chem. 285, 41501–41508. 10.1074/jbc.M110.177600 21030591 PMC3009876

[B71] Sample V. DiPilato L. M. Yang J. H. Ni Q. Saucerman J. J. Zhang J. (2012). Regulation of nuclear PKA revealed by spatiotemporal manipulation of cyclic AMP. Nat. Chem. Biol. 8, 375–382. 10.1038/nchembio.799 22366721 PMC3307945

[B72] Schröder-Lang S. Schwärzel M. Seifert R. Strünker T. Kateriya S. Looser J.. (2007). Fast manipulation of cellular cAMP level by light in vivo. Nat. Methods 4, 39–42. 10.1038/nmeth975 17128267

[B73] Serizawa S. Miyamichi K. Takeuchi H. Yamagishi Y. Suzuki M. Sakano H. (2006). A neuronal identity code for the odorant receptor-specific and activity-dependent axon sorting. Cell 127, 1057–1069. 10.1016/j.cell.2006.10.031 17129788

[B74] Shelly M. Cancedda L. Heilshorn S. Sumbre G. Poo M.-M. (2007). LKB1/STRAD promotes axon initiation during neuronal polarization. Cell 129, 565–577. 10.1016/j.cell.2007.04.012 17482549

[B75] Shelly M. Cancedda L. Lim B. K. Popescu A. T. Cheng P. Gao H.. (2011). Semaphorin3A regulates neuronal polarization by suppressing axon formation and promoting dendrite growth. Neuron 71, 433–446. 10.1016/j.neuron.2011.06.041 21835341 PMC3164872

[B76] Shelly M. Lim B. K. Cancedda L. Heilshorn S. C. Gao H. Poo M. (2010). Local and long-range reciprocal regulation of cAMP and cGMP in axon/dendrite formation. Science 327, 547–552. 10.1126/science.1179735 20110498

[B77] Shewan D. Dwivedy A. Anderson R. Holt C. E. (2002). Age-related changes underlie switch in netrin-1 responsiveness as growth cones advance along visual pathway. Nat. Neurosci. 5, 955–962. 10.1038/nn919 12352982

[B78] Song H. J. Ming G. L. Poo M. M. (1997). cAMP-induced switching in turning direction of nerve growth cones. Nature 388, 275–279. 10.1038/40864 9230436

[B79] Stellwagen D. Shatz C. J. (2002). An instructive role for retinal waves in the development of retinogeniculate connectivity. Neuron 33, 357–367. 10.1016/s0896-6273(02)00577-9 11832224

[B80] Stellwagen D. Shatz C. J. Feller M. B. (1999). Dynamics of retinal waves are controlled by cyclic AMP. Neuron 24, 673–685. 10.1016/s0896-6273(00)81121-6 10595518

[B81] Stierl M. Stumpf P. Udwari D. Gueta R. Hagedorn R. Losi A.. (2011). Light modulation of cellular cAMP by a small bacterial photoactivated adenylyl cyclase, bPAC, of the soil bacterium Beggiatoa. J. Biol. Chem. 286, 1181–1188. 10.1074/jbc.M110.185496 21030594 PMC3020725

[B82] Tang F. Dent E. W. Kalil K. (2003). Spontaneous calcium transients in developing cortical neurons regulate axon outgrowth. J. Neurosci. 23, 927–936. 12574421 10.1523/JNEUROSCI.23-03-00927.2003 PMC6741922

[B83] Terrin A. Di Benedetto G. Pertegato V. Cheung Y.-F. Baillie G. Lynch M. J.. (2006). PGE(1) stimulation of HEK293 cells generates multiple contiguous domains with different [cAMP]: role of compartmentalized phosphodiesterases. J. Cell Biol. 175, 441–451. 10.1083/jcb.200605050 17088426 PMC2064521

[B84] Terrin A. Monterisi S. Stangherlin A. Zoccarato A. Koschinski A. Surdo N. C.. (2012). PKA and PDE4D3 anchoring to AKAP9 provides distinct regulation of cAMP signals at the centrosome. J. Cell Biol. 198, 607–621. 10.1083/jcb.201201059 22908311 PMC3514031

[B85] Tojima T. Itofusa R. Kamiguchi H. (2009). The nitric oxide-cGMP pathway controls the directional polarity of growth cone guidance via modulating cytosolic Ca2+ signals. J. Neurosci. 29, 7886–7897. 10.1523/jneurosci.0087-09.2009 19535600 PMC6665622

[B86] Valsecchi F. Konrad C. Manfredi G. (2014). Role of soluble adenylyl cyclase in mitochondria. Biochim. Biophys. Acta [Epub ahead of print]. 10.1016/j.bbadis.2014.05.035 24907564 PMC4257896

[B87] Vincent P. Castro L. R. V. Gervasi N. Guiot E. Brito M. Paupardin-Tritsch D. (2012). PDE4 control on cAMP/PKA compartmentation revealed by biosensor imaging in neurons. Horm. Metab. Res. 44, 786–789. 10.1055/s-0032-1311631 22581649

[B88] Welker E. Armstrong-James M. Bronchti G. Ourednik W. Gheorghita-Baechler F. Dubois R.. (1996). Altered sensory processing in the somatosensory cortex of the mouse mutant barrelless. Science 271, 1864–1867. 10.1126/science.271.5257.1864 8596955

[B89] Willoughby D. Cooper D. M. F. (2006). Ca2+ stimulation of adenylyl cyclase generates dynamic oscillations in cyclic AMP. J. Cell Sci. 119, 828–836. 10.1242/jcs.02812 16478784

[B90] Willoughby D. Cooper D. M. F. (2007). Organization and Ca2+ regulation of adenylyl cyclases in cAMP microdomains. Physiol. Rev. 87, 965–1010. 10.1152/physrev.00049.2006 17615394

[B91] Willoughby D. Everett K. L. Halls M. L. Pacheco J. Skroblin P. Vaca L.. (2012). Direct binding between Orai1 and AC8 mediates dynamic interplay between Ca2+ and cAMP signaling. Sci. Signal. 5:ra29. 10.1126/scisignal.2002299 22494970

[B92] Willoughby D. Masada N. Wachten S. Pagano M. Halls M. L. Everett K. L.. (2010). AKAP79/150 interacts with AC8 and regulates Ca2+-dependent cAMP synthesis in pancreatic and neuronal systems. J. Biol. Chem. 285, 20328–20342. 10.1074/jbc.m110.120725 20410303 PMC2888445

[B94] Wong W. Scott J. D. (2004). AKAP signalling complexes: focal points in space and time. Nat. Rev. Mol. Cell Biol. 5, 959–970. 10.1038/nrm1527 15573134

[B93] Wong S. T. Trinh K. Hacker B. Chan G. C. Lowe G. Gaggar A.. (2000). Disruption of the type III adenylyl cyclase gene leads to peripheral and behavioral anosmia in transgenic mice. Neuron 27, 487–497. 10.1016/s0896-6273(00)00060-x 11055432

[B95] Wu K. Y. Zippin J. H. Huron D. R. Kamenetsky M. Hengst U. Buck J.. (2006). Soluble adenylyl cyclase is required for netrin-1 signaling in nerve growth cones. Nat. Neurosci. 9, 1257–1264. 10.1038/nn1767 16964251 PMC3081654

[B96] Zaccolo M. Movsesian M. A. (2007). cAMP and cGMP signaling cross-talk: role of phosphodiesterases and implications for cardiac pathophysiology. Circ. Res. 100, 1569–1578. 10.1161/circresaha.106.144501 17556670

[B97] Zhang J. Ackman J. B. Xu H.-P. Crair M. C. (2012). Visual map development depends on the temporal pattern of binocular activity in mice. Nat. Neurosci. 15, 298–307. 10.1038/nn.3007 22179110 PMC3267873

[B98] Zhang J. Ma Y. Taylor S. S. Tsien R. Y. (2001). Genetically encoded reporters of protein kinase A activity reveal impact of substrate tethering. Proc. Natl. Acad. Sci. U S A 98, 14997–15002. 10.1073/pnas.211566798 11752448 PMC64972

[B99] Zippin J. H. Chen Y. Nahirney P. Kamenetsky M. Wuttke M. S. Fischman D. A.. (2003). Compartmentalization of bicarbonate-sensitive adenylyl cyclase in distinct signaling microdomains. FASEB J. 17, 82–84. 10.1096/fj.02-0598fje 12475901

[B100] Zou D.-J. Chesler A. T. Le Pichon C. E. Kuznetsov A. Pei X. Hwang E. L.. (2007). Absence of adenylyl cyclase 3 perturbs peripheral olfactory projections in mice. J. Neurosci. 27, 6675–6683. 10.1523/jneurosci.0699-07.2007 17581954 PMC6672705

